# Polymorphisms of Codons 110, 146, 211 and 222 at the Goat *PRNP* Locus and Their Association with Scrapie in Greece

**DOI:** 10.3390/ani11082340

**Published:** 2021-08-08

**Authors:** Athanasios I. Gelasakis, Evridiki Boukouvala, Maria Babetsa, Efstathios Katharopoulos, Vayia Palaska, Dimitra Papakostaki, Nektarios D. Giadinis, Dimitrios Loukovitis, Jan P. M. Langeveld, Loukia V. Ekateriniadou

**Affiliations:** 1 Department of Animal Science, School of Animal Biosciences, Agricultural University of Athens, 11855 Athens, Greece; gelasakis.vet@gmail.com; 2 Veterinary Research Institute, ELGO-DIMITRA, 54124 Thessaloniki, Greece; boukouvala@vri.gr (E.B.); mmpampet@bio.auth.gr (M.B.); ekatharo@gmail.com (E.K.); 3 National Reference Laboratory for TSEs, Ministry of Agricultural Development and Food, 41110 Larissa, Greece; tsevetlab@hotmail.com; 4 Veterinary Center of Thessaloniki, Ministry of Agricultural Development and Food, 54627 Thessaloniki, Greece; viroltse@gmail.com; 5 School of Veterinary Medicine, Aristotle University of Thessaloniki, 54627 Thessaloniki, Greece; ngiadini@vet.auth.gr; 6 Research Institute of Animal Science, ELGO-DIMITRA, 58100 Giannitsa, Greece; dloukovi@hotmail.com; 7 Department of Infection Biology, Wageningen Bioveterinary Research (WBVR), 8221 RA Lelystad, The Netherlands; jan.langeveld@wur.nl

**Keywords:** scrapie, goats, polymorphisms, genetic resistance, breeding strategies

## Abstract

**Simple Summary:**

Scrapie is a transmissible spongiform encephalopathy affecting sheep and goats. Due to similarities with mad cow disease (BSE) which is transmissible to humans, scrapie is considered of significance for its theoretical zoonotic potential. A variety of control strategies have been proposed and applied over the years. While herd depopulation, culling of affected susceptible animals, and disinfection measures are poorly successful, selective breeding schemes focused on amino acid substitutions (polymorphisms) in prion protein (PrP) would be the best way for eradication. In sheep, the association between PrP gene polymorphism argininine on codon 171 is highly effective for scrapie resistance breeding. Although in goats this polymorphism is absent, three other candidate PrP amino acid polymorphisms have been recognized for resistance breeding. For Greece with the largest goat population in Europe, the frequencies of these polymorphisms has yet to be sufficiently documented. This study performed a large-scale, cross-sectional survey for a large part of the country, regarding PrP polymorphic codons 110, 146, 211, and 222, and estimated effects of these polymorphisms on (i) the likelihood to be infected by scrapie, and (ii) scrapie status at the herd level, revealing possible candidate PrP amino acid polymorphisms to be used in breeding for scrapie-resistance programs.

**Abstract:**

Scrapie is considered an endemic disease in both sheep and goats in Greece. However, contrary to sheep, in goats more than one prion protein (PrP) polymorphism has been recognized as a candidate for resistance breeding against the disease. For an impression, candidates which are circulating, (i) brain samples (*n =* 525) from scrapie-affected (*n =* 282) and non-affected (*n =* 243) animals within the national surveillance program, and (ii) individual blood samples (*n =* 1708) from affected (*n =* 241) and non-affected (*n =* 1467) herds, in a large part of mainland Greece and its islands, were collected and assayed. A dedicated Taqman method was used to test for amino acid polymorphisms 110T/P, 146N/S/D, 211R/Q, and 222Q/K. Highly prevalent genotypes were 110TT, 146NN, 211RR, and 222QQ. The frequencies of polymorphisms in blood and negative brain samples for codons 110P, 211Q, and 222K were 4.0%, 3.0%, and 1.9%, respectively, while 146D (0.7%) was present only on Karpathos island. Codon 110P was exclusively found in scrapie-negative brains, and homozygous 110P/P in two scrapie-negative goats. It is concluded that breeding programs in Karpathos could focus on codon 146D, while in other regions carriers of the 110P and 222K allele should be sought. Case-control and challenge studies are now necessary to elucidate the most efficient breeding strategies.

## 1. Introduction

Dairy goat farming in the EU ranks among the dynamic and promising livestock sectors, growing fast to satisfy the increasing demand for goat milk and dairy products [1]. Its multidimensional socio-economic, cultural, and environmental role in different regions across Europe has led to a gradation of the farming systems from low-input, traditional systems to high-input, intensive ones. Consequently, case-specific, and evidence-based adjustments on production methods and management schemes are necessary, although scarce. Scarcity of the aforementioned adjustments impacts the health status at the herd level and facilitates the emergence of new diseases or modifications in the epizootiology and transmission dynamics of the existing ones. 

Scrapie is among these diseases and in Greece has been diagnosed since 1987 in sheep and 1997 in goats [2]. It is a significant and notifiable disease since though its zoonotic potential is supposedly low, it has close similarities with the zoonotic and epizootic bovine spongiform encephalopathy (BSE) [3]. Scrapie is a chronic, neurodegenerative prion disease that affects both sheep and goats, usually around 2 to 5 years of age, and can be fatal for the affected animals. At the first stages of the disease, mild behavioral changes occur, which later develop into neurological disease with hyperesthesia, ataxia, and pruritus. During the last stages, signs of systemic disease are prevalent, including loss of body weight, anorexia, lethargy, and death. It was first reported in goats by Chelle [4] and since then, clinical cases of scrapie have been recorded in many countries [5,6]. The main constituent of the infectious agent is an aberrant isoform (PrP^Sc^) of the normal cellular (PrP^C^) prion protein (PrP), which is a cell–surface glycoprotein. The transmission and the incubation period of the disease depend on the exposure to the infectious agent, the scrapie strain, and the genetic background of the host [7]. In sheep, the polymorphisms at codons 136, 154, and 171 are known to be closely linked with susceptibility to natural and experimental scrapie, which have been utilized in the European countries for the implementation of national breeding programs to control scrapie. It is also known that scrapie occurs in two forms, namely the classical and the atypical one, which differ in their incubation period, clinical signs, neuropathological lesion profiles, and/or PrP^Sc^ biochemical properties. Control strategies which have been utilized over the years for small ruminants include: (i) Complete herd depopulation, (ii) depopulation of genetically related animals (maternal transmission), (iii) depopulation of animals at risk, (iv) culling of affected animals, (v) cleaning and disinfection of premises and equipment, (vi) sufficient period until animals will be restocked in the premises, and (vii) selective breeding schemes based on the PrP gene polymorphisms (only for classical scrapie) [8,9]. Although scrapie is commonly found in goats reared in mixed sheep-goat flocks, it does also occur within goat herds [10,11,12].

Greece has the largest national goat flock in Europe with more than 2.6 × 10^6^ milking goats (ca. 30% of the EU milking goat herd) producing about 356,000 tons of milk (ca. 15% of the total EU goat milk production), mainly used to produce Feta cheese [1]. A remarkable diversity of farming systems is present depending on the diverse landscape and natural sources availability across the country. In semi-mountainous and mountainous areas of the mainland and the islands, semi-extensive, low-input farming systems are the most prevalent, whereas, in lowlands, semi-intensive and intensive systems are mainly observed. Most goats belong to indigenous Greek breeds, however, bucks of foreign breeds have been extensively, but not systematically, used in the past as breeding stocks and exclusively for the improvement of milk production traits. Breeding for resistance against diseases is not a common practice and control of critical diseases is mainly based on national control and eradication programs or the prevention at the herd level. Scrapie is diagnosed in both sheep flocks and goat herds and national programs for the control and eradication of classical scrapie have been elaborated and implemented for both species. Until 2015, more than 50 sheep and goat flocks/herds have been culled in Greece. However, during the last years and due to the increasing exploitation of resistant rams (ARR/ARR) in breeding for resistance programs, and culling of affected flocks/herds, a decline in new cases of scrapie is observed.

In sheep, selective breeding schemes have been exploited, to eliminate the prevalence of scrapie in the EU, based on the selection of a resistance-related polymorphism in the *PRNP* gene that is coding for the prion protein named PrP (the so called ARR allele of PrP, EC 999/2001) [13]. Several polymorphisms are known that determine disease incubation time, as well as the molecular mechanism of the PrP^C^ conversion into the pathogenic isoform PrP^Sc^ [4,5,14,15,16,17,18,19,20,21,22]. The success of nation-wide application of these breeding programs is evident by the remarkable increase (more than 28% in 4 years) of the scrapie-resistant ARR alleles, which has been observed after the implementation of these programs [10] and exemplified in the country’s noticeable reduction of the disease prevalence in sheep breeds [23]. 

Similarly to sheep, goat PrP is very polymorphic, but the critical arginine at codon 171 used in sheep for scrapie-resistance breeding does not occur in goats [5,19,24,25,26,27,28,29]. Furthermore, more than 50 *PRNP* gene polymorphisms have been found, however, their role regarding the incubation period and the susceptibility to classical scrapie has not been adequately documented. Atypical scrapie has also been detected in goats in many countries including Switzerland, France, Spain, and Italy. Cases of atypical scrapie are distinguished by a 5-band profile on Western blot with a prominent lower band at approximately 11 to 12 kDa, whereas, classical scrapie has the nonglycosylated band at approximately 19 to 21 kDa. In addition, the PrP^Sc^ of atypical scrapie is relatively sensitive to proteinases [30,31]. In the last 15 years, many polymorphisms associated with resistance/susceptibility to scrapie, such as 110T/P, 127G/S, 142M/I, 145G/D, 146N/D/S, 154R/H, 211R/Q, and 222Q/K have been described in different breeds of goats and regions [5,32,33,34,35,36,37,38,39,40]. Based on these data and experimental challenge studies, the European Food Safety Authority (EFSA) has assigned the polymorphisms 222K, 146D, and 146S as acceptable candidates for breeding towards resistance against scrapie [13,41]. However, the frequency of these candidate alleles is usually low and has not yet been sufficiently documented in Greece (as well as in most regions worldwide) to undertake an evidenced-based breeding strategy.

Estimating the frequency of candidate resistant alleles and quantifying their effect is a preliminary and crucial step in determining the feasibility, impact, and cost-effectiveness of the proposed selective breeding programs [42]. The objective of this study was 2-fold. Firstly, we performed a large-scale, cross-sectional survey regarding the existence of potentially scrapie-resistant polymorphisms at 110, 146, 211, and 222 codons, in Greece (mainland and selected islands). Secondly, we estimated the effects of these polymorphisms on the (i) likelihood of a goat to be infected by scrapie, and (ii) the herd’s scrapie status. Additionally, we assess and discuss possible candidate genotypes to be used in breeding for scrapie-resistance programs.

## 2. Materials and Methods

### 2.1. Animals and Sample Collection

**Figure 1:**
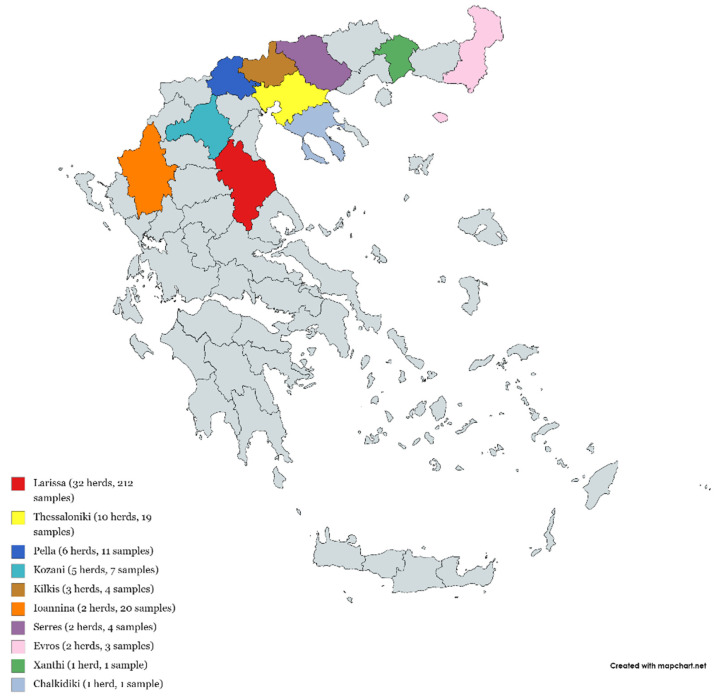
Distribution of the 64 herds and 282 brain samples found positive for scrapie.

A total of 282 and 243 brain samples from scrapie-affected (64 herds) and non-affected (11 herds) animals were collected, respectively, within the national TSEs surveillance program between years 2008–2014 and analyzed to detect polymorphisms at the *PRNP* gene locus. Brain sampling took place according to the European Parliament and Council Regulation EC no. 999/2001 [13] from the suspected animals’ brainstem, either from affected or from non-affected herds. All samples were collected once from each individual herd involved in the study over the 7-year period and were analyzed and confirmed as positive or negative by biochemical and immunohistochemical techniques (Bio-Rad TeSeE SAP rapid test, Bio-Rad TeSeE Sheep/Goat rapid test, IDEXX HerdChek BSE-Scrapie Antigen Test Kit, EIA, Prionics Check PrioSTRIP SR) (Figure 1). The number of samples per herd varied and was decided on a case-specific basis depending on the suspected herd scrapie status. 

**Figure 2:**
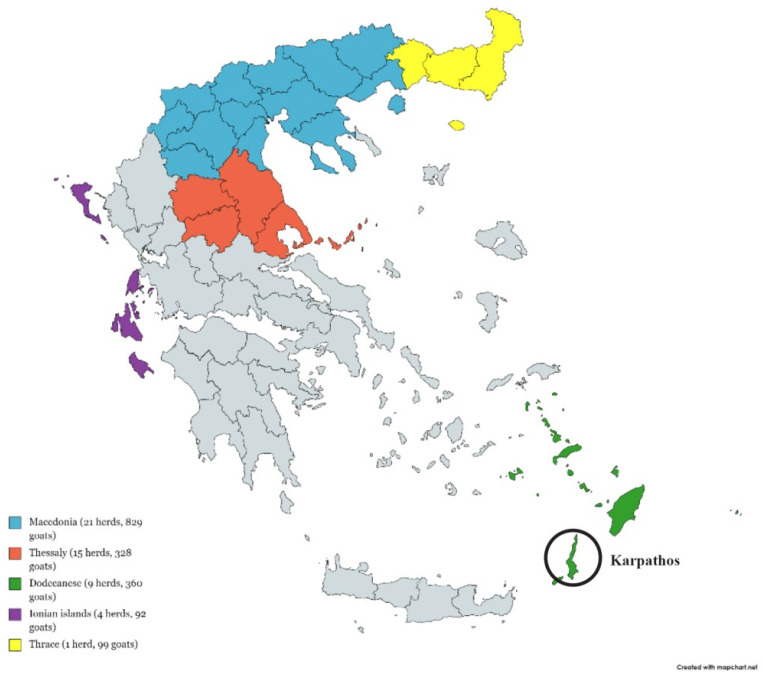
Distribution of the 50 herds and 1708 goats, where blood samples were collected.

Blood samples were collected randomly from affected and non-affected herds, as a part of the standard veterinary clinical practice and were assayed within the EU EMIDA ERA-NET project entitled “Towards breeding of goats for genetically determined TSE resistance”. A total of 1708 blood samples was collected during a single farm visit from individual, clinically healthy goats reared in affected (*n =* 12 herds, 241 samples) and non-affected (*n =* 38 herds, 1467 samples) herds. Enrolled herds were in the prefectures of Macedonia and Thessaly, Dodecanese and Ionian Islands, and Thrace (Figure 2).

### 2.2. Genomic DNA Extraction and Single-Nucleotide Polymorphisms (SNPs) Detection

From all samples (brain and blood), genomic DNA was extracted using the PureLink Genomic DNA Mini Kit (Life Sciences) according to the manufacturer’s instructions. The *PRNP* coding region was amplified with a newly designed method of Taqman probes for polymorphisms 110T/P, 146N/S/D, 211R/Q, and 222Q/K [43], oligonucleotides are listed in Table 1. All samples were assayed for 146N/S/D, 211R/Q, and 222Q/K, whereas, a subtotal of 449 brain samples (including all the scrapie-positive samples) and 968 blood samples (including all the samples from scrapie-positive herds) were assayed for 110T/P. Amplification reaction mixtures were prepared at a final volume of 12.5 μL containing 1X KAPA2G PROBE FAST qPCR Master Mix (KAPA BIOSYSTEMS), 400 nM of each primer and probes, and 80–100 ng of the sample’s DNA. The qPCRs were performed in a StepOnePlus Real Time PCR System (AB Applied Biosystems). The cycling conditions for all the reactions consisted of the initial denaturation at 95 °C for 3 min, 45 cycles of denaturation at 95 °C for 3 s, and annealing/extension at 62 °C for 30 s. The SNPs detection method was validated using as controls goat genomic DNA samples with all possible genotypes at codons 110, 146, 211, and 222, and a selected number of the animals in the study (*n =* 80), which were sequenced by the Sanger method in an ABI 3500 Genetic Analyzer (Applied Biosystems) performing direct sequencing of the full coding region. 

### 2.3. Statistical Analyses 

Allelic and genotypic frequencies were calculated with counting. Allelic frequencies were used to calculate the expected numbers of genotypes under the assumed Hardy-Weinberg equilibrium. The observed and expected numbers of genotypes were compared using the chi-square test as described below:

$$\chi^{2}=\sum \frac{{\left(o-e\right)}^{2}}{e}$$

where *o* is the observed and *e* is the expected number of each genotype assuming the Hardy-Weinberg equilibrium. The effects of the 146N/S/D, 211R/Q, and 222Q/K polymorphisms (i) on the herd’s scrapie-status (blood sample analysis), and (ii) on the animal’s scrapie-status (brain sample analysis) were assessed using the following binary regression model:

S_jkl_ = m + A_j_ + B_k_ + C_l_ + e_jkl_

where S is the herd’s scrapie status (0 is the absence of scrapie at the herd level, 1 is the presence of scrapie at the herd level) or the animal’s scrapie status (0 is the absence of scrapie at the animal level, 1 is the presence of scrapie at the animal level), A is the fixed effect of jth genotype 146 (five levels, N/N, N/S, N/D, S/S, and D/D), B is the fixed effect of kth genotype 211 (two levels, R/R and R/Q), C is the fixed effect of lth genotype 222 (two levels, Q/Q and Q/K), and e is the random residual. Polymorphisms 110 (T/P) were not introduced in the model. The SPSS v23 software (IBM Corp., Armonk, NY, USA) was used for the statistical analyses and the significance level was set at the 0.05 level. 

### 2.4. Ethics Statement

Blood and brain samples were either routinely collected for the interest of the national scrapie eradication program or as a part of the non-experimental standard clinical veterinary practice. In any case, this study followed the European Directive 86/609/EEC and its national implementation by the Greece Presidential Decree no. 160/1991 (Governmental Gazette no. A’ 64).

## 3. Results

Frequencies of genotypes and alleles at codons 110, 146, 211, and 222 for (i) brain samples from scrapie-positive and scrapie-negative goats, and (ii) blood samples from scrapie-positive and scrapie-negative herds are summarized in Table 2. In scrapie-positive brain samples, the frequencies of genotypes 110TT, 146NN, 211RR, and 222QQ were 100% (282/282), 99.6% (281/282), 95.7% (270/282), and 95.4% (269/282), respectively, whereas, genotypes 110TP, 110PP, 146ND, 146DD, and 146SS were not found in any case. In these samples, the most prevalent alleles were 110T (100.0%, 564/564), 146N (99.8%, 563/564), 211R (97.9%, 552/564), and 222Q (97.7%, 551/564). Genotypes, 211QQ and 222KK were not found in any of the samples analyzed (brain or blood). A similar pattern regarding the frequencies of genotypes and alleles was observed for scrapie-negative brain samples and for goats from both scrapie-positive and scrapie-negative herds (Table 2). Namely, frequencies of genotypes 110TT, 146NN, 211RR, and 222QQ were 94.2% (227/241), 97.5% (235/241), 81.7% (197/241), and 96.3% (232/241), respectively for scrapie-positive herds, and 91.1% (662/727), 95.0% (1394/1467), 95.8% (1406/1467), and 96.7% (1418/1467), respectively, for scrapie-negative herds. Moreover, the predominant alleles were 110T, 146N, 211R, and 222Q in both scrapie-positive [97.1% (468/482), 98.8% (476/482), 90.9% (438/482), and 98.1% (473/482), respectively] and scrapie-negative herds [95.4% (1387/1454), 97.4% (2858/2934), 97.9% (2873/2934), and 98.3% (2885/2934), respectively]. For the first time, genotype 110PP was found in Greek goats in two animals from the group of scrapie-negative herds (0.3%, 2/727), whereas, 110TP was not found in any case in scrapie-positive animals, although it was found in scrapie-negative animals, as well as in goats from both scrapie-positive and scrapie-negative herds. At codon 146, all possible polymorphisms have been detected. Genotypes at this codon, detected for the first time in Greek goats, were 146ND (26 goats (1.8%, 26/1467)), 146SS, and 146DD [at low frequencies, 0.1% (2/1467) and 0.1% (1/1467), respectively] all originating from the island of Karpathos and only from the group of scrapie-negative herds. The studied goat population was at the Hardy-Weinberg equilibrium with respect to codons 110, 211, and 222 but not to codon 146 for both scrapie-positive and scrapie-negative herds.

Table 3 summarizes the frequencies of polymorphisms at codons 146, 211, and 222 of the PRNP gene and the effects of the observed genotypes (i) on individual goats’ scrapie-status (brain samples), and (ii) on the herds’ scrapie-status (blood samples), as derived by the regression models. In detail, the model used to assess the main effects of genotypes on the goat scrapie-status did not produce any significant results (Table 3). However, a clear tendency was observed by the 146NS genotype to be protective against scrapie, namely, the probability of scrapie infection was 7.1 times lower for 146NS compared to 146NN (*p* = 0.065, 95% CI, 0.9–50.0). Moreover, the 211RQ genotype was ca. 5.6 times more likely to be observed in scrapie-positive herds when compared to goats with the 211RR genotype (*p* < 0.001, 95% CI, 3.7–8.6), whereas, frequencies of polymorphisms at codons 146 and 222 were not found to significantly differ between scrapie-positive and scrapie-negative herds.

## 4. Discussion

Attempts to propose breeding for scrapie resistance programs in Greece have been made by Vouraki et al. [43] and Kanata et al. [44]. However, their proposals were based on studies involving only clinically healthy goats from scrapie-negative herds (436 and 766 goats, respectively), targeting on 211Q, 146S, and 222K alleles as being the most promising resistant alleles. To address the demand of such programs, our study focused on four polymorphisms of interest in caprine PrP, encompassing a large-scale survey of both affected and non-affected goats (525 brain samples) and herds (1708 blood samples), which can be considered of general interest since it was performed in Greece with the highest number of goats in Europe. In our study, the results suggested continuing on the 222K allele, 146S, 146D, and yielded a new promising codon which is 110P.

At codon 110, genotype 110TP was found in both scrapie-negative and scrapie-positive farms and in scrapie-negative animals but in no case in scrapie-positive animals. This is a strong indication of the protective effect of genotype 110TP, though it was not possible to be statistically proven, as the effect of codon 110 on scrapie status was not estimated by the model. In any case, further testing in experimental challenge studies is necessary to confirm this assumption. If genotype 110TP is proved to exhibit a protective action against scrapie its inclusion in EU legislation needs to be supported by EFSA. Moreover, it is the first time that the homozygous genotype 110PP is observed in goats in Greece (two animals, both from scrapie-negative farms). 

Due to the absence or very low frequencies of alleles D146 and S146 in Europe, except from Cyprus, no results from field studies regarding resistance of genotypes at codon 146 to European scrapie strains have been available up to now [45]. This has led EFSA to conclude that more epidemiological studies were needed in the case of codon 146. In our study, this demand has been addressed, as polymorphisms at the codon 146 were found and assessed. In particular, genotype 146ND was observed in 1.5% of the goats originating exclusively from the scrapie-negative herds group (26/1467). Therefore, a comparison between 146ND and 146NN (reference category) in regards to scrapie occurrence was not possible to produce a valid odds ratio. It is likely that the enrolment of more animals with the NS genotype could have revealed a measurable, significant resistance potential for 146ND. No other genotypes were found to significantly affect the goat scrapie-status, although a tendency for the 146NS genotype to be protective against scrapie was evidenced. The likelihood of a goat being infected by scrapie was ca. 7 times less for 146NS compared to 146NN (*p* = 0.065). These findings are in accordance with the available literature, where a lower susceptibility of both 146ND and 146NS genotypes and a higher genotype for 146NN to natural and experimental scrapie infection have been observed in field studies [5,22,28,32,35]. The latter observation has been experimentally confirmed by Niedermeyer et al. [46] and increased (i) resistance of genotypes 146DD, 146SS, 146ND, and 146NS, and (ii) incubation period of genotypes 146DD, 146SS, and 146NS, compared to 146NN have been documented. These findings had justified the proposed strategy to exploit codon 146 polymorphisms in selection breeding schemes for the control and eradication of classical scrapie in Cyprus [46]. On the contrary, at the herd level, polymorphisms at codon 146 were not related to the likelihood of a goat to originate from a scrapie-positive or scrapie-negative herd. Furthermore, genotypes 146SS and 146DD were found for the first time in Greek goats. However, at low frequencies (0.2% and 0.1%, respectively) which did not allow valid comparisons with 146NN to reveal a possible protective or susceptibility potential. Nonno et al. [45] studied several goat isolates using rodent lines for strain-type-bioassaying, showing that Greek scrapie strain features compared well to Cypriotic. Therefore, it can be assumed that the variants at codon 146 may be efficient against Greek isolates.

In the case of codon 211, it was found that goats with the 211RQ genotype were ca. 5.6 times more likely to originate from a scrapie-positive herd when compared to goats with the 211RR genotype (*p* < 0.001). It could be assumed that the 211RQ genotype may be associated with susceptibility to the disease at the herd level. However, this assumption is not supported by the model used to compare genotypes at the animal level, where no differences were observed. Additionally, it is not supported by the available literature. For example, Barillet et al. [33] have concluded that the 211RQ genotype was resistant against scrapie in Alpine and Saanen goats. In our study, it is likely that the increased frequency of 211RQ genotypes in scrapie-positive herds compared to scrapie-negative ones (18.3% vs. 4.4%) could have resulted from selective pressure towards more resistant genotypes. However, this is an assumption which cannot be proved, under the current experimental design. Genotype 211QQ could not be assessed in terms of resistance or susceptibility as it was not found in any case.

Field and surveillance data regarding scrapie occurrence and the evaluation of candidate alleles that confer resistance/susceptibility to the disease, have indicated that the 222K allele is associated with strong resistance against it across Europe [31,33,34,35,38,47,48,49,50]. This has been experimentally confirmed by studies where 222Q-Tg501 and 222K-Tg516 mice were challenged by the prion. These experiments showed that 222K-Tg516 mice were resistant to all inoculates, while heterozygous 222QK mice were clearly more resistant to various scrapie isolates compared to 222Q-Tg501 mice, concluding that the 222K allele provides a dominant negative effect over the wild-type PrP sequence [51]. However, in our study, polymorphisms at codon 222 were not found to affect the likelihood of a goat to be infected by scrapie or originate from a scrapie-positive herd. This is not the first time that the protective effect of 222K allele in goat populations is not as strong compared to R171 in sheep, as in the past, a few cases of natural scrapie infections have been reported in heterozygous 222K goats [33,35,44], indicating that a single copy of 222K allele does not confer absolute resistance. In particular, in Greece, goats carrying the 222K allele originating from three scrapie-infected herds in endemic regions were found scrapie-positive [35]. Similarly, Corbiere et al. [49] found scrapie-positive, 222K-carrier goats in three different geographical areas (five infected herds) in France. In both cases, the scarcity of detailed farm characteristics and historical data does not allow proposing a conclusive explanation of this finding. Though, in the case of Greece, it could be assumed that in these farms 222K heterozygous goats were either subjected to a high infectious pressure or were not protected from scrapie infection, due to the involvement of local scrapie strains [35,45]. However, this might not be the case, as it has been evidenced (by the low 222K PrP in the prion materials) that it is not really a PrP-variant that gets involved in prion formation [52]. Additionally, the level of PrP^res^ measured in the past, in Greek brain samples was exceptionally low (G11 211RQ, 222QK <1.6 ng/mg tissue PrP^res^, G12 222QK <2 ng/mg tissue PrP^res^), which is indicative for a rather high resistance of 222K to PrP^C^>PrP^Sc^ conversion [53]. In our study, a higher prevalence of scrapie in 222K carriers was found when compared to French goats, although there was no evidence from the *PRNP* gene sequences that the 222K allele contains mutations associated with susceptibility. Moreover, the fact that (i) all samples derived from mixed flocks with a high prevalence of sheep scrapie, indicating a strong infection pressure, (ii) the existence of the disease for more than 2 years, and (iii) the potential occurrence of different scrapie strains, need to be considered as significant risk factors and possible confounders. Another study design using farm rather than animal as a statistical unit might address this issue. Moreover, an accurate case-control study in important goat dairy production regions is still required to confirm the quality of the 222K codon.

In our study, no homozygous 222K goats were found to estimate the potential protective effect of the genotype 222K. In general, the 222K allele occurs in many dairy goat breeds, but at rather low frequencies in most of the European countries, e.g., only 0.6–0.7% in different breeds [54]. This is also the case for Greek indigenous breeds [43]. The allele 222K heterogeneous distribution across the EU countries and among different goat breeds, and its low frequency in the majority of cases (<10%), imply that high genetic selection pressure could have an adverse effect on genetic diversity. Therefore, breeding for resistance to TSE programs should be individualized, and 110P, 146S, and 146D alleles should be considered on an evidential basis, following (i) an appropriate epizootiological investigation of the disease, (ii) identification of candidate PrP gene polymorphic alleles that may confer susceptibility or resistance to goat scrapie, (iii) genetic profiling of the existed population, and (iv) assessment of potential impacts on the productivity, health, and welfare status of animals. This explains the reason why although the 222K allele is currently prioritized as the most promising genetic target in selective breeding protocols towards scrapie resistance, a program to efficiently utilize it for the control of scrapie in goats is yet to be implemented. In 2017, EFSA consulted the European Commission to update the current knowledge concerning genetic resistance to TSE in goats. An evaluation tool assessing both the weight of evidence and the level of resistance of the nine most studied candidate alleles was developed, based on the results from field observations and experimental data. Exploitation of the tool has led to the conclusion that K222, D146, and S146 alleles confer adequate genetic resistance against classical scrapie strains, with K222 presenting the highest weight of evidence, and therefore suggested as the priority genetic solution against scrapie. In any case, the EFSA recommendation [29,41] is that selective breeding programs towards scrapie resistance should be developed and managed independently within each country and according to the established frequencies of resistance-associated alleles for each breed. 

## 5. Conclusions

Based on our findings and the results from relevant studies in Greece, it could be concluded that breeding programs could be based on the codon 146 for Karpathos island and 222 for the mainland. Codon 110 needs to be also considered as a promising one, however more evidence regarding its performance need to be acquired. Furthermore, codon 146D could also be included in these inventory studies [38]. Therefore, choosing regional suitable alleles for resistance breeding against scrapie in goats remains a challenging research area [40,54,55]. Several actions need to be taken at this stage in Greece to accomplish a scrapie-resistant goat population, namely (i) to produce regional polymorphism inventories for areas of Greece not yet explored, (ii) further case-control studies, (iii) challenge experiments in transgenic mice with Greek isolates to confirm the quality of the 222K allele for resistance breeding, and (iv) organizing breeding centers for 146D, 146S, 110P, and 222K goats.

## Figures and Tables

**Table 1:** Primers and probes sequences used in TaqMan SNP genotyping assays for the detection of polymorphisms 110 (T/P), 146 (N/S), 146 (N/D), 211 (R/Q), and 222 (Q/K).

Codon for Single Nucleotide Polymorphism (SNP)	Primer	
	Forward	Reverse
110(T/P)	TCAGTGGAACAAGCCCAGTAAG	AGCAGCTCCTGCCACATG
146(N/S)	GCCATGAGCAGGCCTCTTATA	GGGTAACGGTACATGTTTTCACGAT
146(N/D)	GCCATGAGCAGGCCTCTT	GGGTAACGGTACATGTTTTCACGAT
211(R/Q)	GAACTTCACCGAAACTGACATCAAG	ACTGGGTGATGCACATTTGCT
222(Q/K)	TGGTGGAGCAAATGTGCATCA	GGGAAGAAAAGAGGATCACACTTG
	Probe *	
	**FAM**	**VIC^®^**
110(T/P)	TCATGTTGGGTTTTGG	CTTCATGTTGGTTTTTGG
146(N/S)	TTTTGGCAGTGACTATG	CATTTTGGCAATGACTATG
146(N/D)	CATTTTGGCAATGACT	ATACATTTTGGCGATGACT
211(R/Q)	AATGGAGCAAGTGGTG	ATAATGGAGCGAGTGGTG
222(Q/K)	CTGGGATTCTCTCTTGTACTG	TGGGATTCTCTCTGGTACTG

* FAM: 6-Carboxyfluorescein; VIC: 2-Chloro-7 phenyl-1,4-dichloro-6-carboxy-fluorescein.

**Table 2:** Frequencies of genotypes and alleles at codons 110, 146, 211, and 222 for (i) scrapie-positive and scrapie-negative brain samples, and (ii) for goats from scrapie-positive and scrapie-negative herds.

		Brain Samples		Blood Samples	
		I. Scrapie-Positive	II. Scrapie-Negative	III. Scrapie-Positive Herds	IV. Scrapie-Negative Herds
		Count (%)	Count (%)	Count (%)	Count (%)
Codon 110 *		*n* = 282	*n =* 167	*n =* 241	*n =* 727
Genotype	TT	282 (100.0)	158 (94.6)	227 (94.2)	662 (91.1)
	TP	0 (0.0)	9 (5.4)	14 (5.8)	63 (8.7)
	PP	0 (0.0)	0 (0.0)	0 (0.0)	2 (0.3)
Allele	T	564 (100.0)	325 (97.3)	468 (97.1)	1387 (95.4)
	P	0 (0.0)	9 (2.7)	14 (2.9)	67 (4.6)
Codon 146		*n =* 282	*n =* 243	*n =* 241	*n =* 1467
Genotype	NN	281 (99.6)	237 (97.5)	235 (97.5)	1394 (95.0)
	ND	0 (0.0)	0 (0.0)	0 (0.0)	26* (1.8)
	NS	1 (0.4)	6 (2.5)	6 (2.5)	44 (3.0)
	DD	0 (0.0)	0 (0.0)	0 (0.0)	1 (0.1)
	SS	0 (0.0)	0 (0.0)	0 (0.0)	2 (0.1)
	DS	0 (0.0)	0 (0.0)	0 (0.0)	0 (0.0)
Allele	N	563 (99.8)	480 (98.8)	476 (98.8)	2860 (97.5)
	S	1 (0.2)	6 (1.2)	6 (1.2)	46 (1.6)
	D	0 (0.0)	0 (0.0)	0 (0.0)	28 (0.9)
Codon 211 *		*n =* 282	*n =* 243	*n =* 241	*n =* 1467
Genotype	RR	270 (95.7)	230 (94.7)	197 (81.7)	1406 (95.8)
	RQ	12 (4.3)	13 (5.3)	44 (18.3)	61 (4.2)
	QQ	0 (0.0)	0 (0.0)	0 (0.0)	0 (0.0)
Allele	R	552 (97.9)	473 (97.3)	438 (90.9)	2873 (97.9)
	Q	12 (2.1)	13 (2.7)	44 (9.1)	61 (2.1)
Codon 222 *		*n =* 282	*n =* 243	*n =* 241	*n =* 1467
Genotype	QQ	269 (95.4)	226 (93.0)	232 (96.3)	1418 (96.7)
	QK	13 (4.6)	17 (7.0)	9 (3.7)	49 (3.3)
	KK	0 (0.0)	0 (0.0)	0 (0.0)	0 (0.0)
Allele	Q	551 (97.7)	469 (96.5)	473 (98.1)	2885 (98.3)
	K	13 (2.3)	17 (3.5)	9 (1.9)	49 (1.7)

* Codons at the Hardy-Weinberg equilibrium state in the studied goat population (*p* > 0.01).

**Table 3:** Genotypic frequencies (%) and corresponding number of animals (in parenthesis) for codons 146, 211, and 222 of the PRNP gene, as well as the effects of the observed genotypes (i) on individual goat scrapie-status (brain samples), and (ii) on the herd scrapie-status (blood samples).

**Brain Samples (*n =* 525)**								
**Codon**	**Genotype**	**% (Number of Animals)**	**B**	**SE**	***p*-Value**	**Odds Ratio**	**95% CI**	
							**Lower**	**Upper**
146	NS	1.3 (7)	−2.00	1.08	0.065	0.14	0.02	1.13
	NN	98.7 (518)	*Ref.*					
211	RQ	4.8 (25)	−0.27	0.41	0.507	0.76	0.34	1.70
	RR	95.2 (500)	*Ref.*					
222	QK	5.7 (30)	−0.47	0.38	0.216	0.63	0.30	1.32
	QQ	94.3 (495)	*Ref.*					
		Constant	0.21	0.09	0.024	1.24	-	-
**Blood samples (*n =* 1708)**								
**Codon**	**Genotype**	**% (Number of animals)**	**B**	**SE**	***p*-value**	**Odds ratio**	**95% CI**	
							**Lower**	**Upper**
146	DD	0.1 (1)	NV					
	SS	0.1 (2)	NV					
	ND	1.5 (26)	NV					
	NS	2.9 (50)	−0.16	0.45	0.716	0.85	0.35	2.05
	NN	95.4 (1629)	*Ref.*					
211	RQ	6.1 (105)	1.73	0.22	0.000	5.62	3.68	8.59
	RR	93.9 (1603)	*Ref.*					
222	QK	3.4 (58)	0.17	0.38	0.659	1.18	0.57	2.46
	QQ	96.6 (1650)	*Ref.*					
		Constant	−1.95	0.08	0.000	0.14	-	-

B: Regression coefficient; SE: Standard error; CI: Confidence interval; *Ref*.: Reference category.

## Data Availability

The data used in this study are available on request from the corresponding author.
